# Clinicopathological and prognostic value of S100A4 expression in non-small cell lung cancer: a meta-analysis

**DOI:** 10.1042/BSR20201710

**Published:** 2020-07-30

**Authors:** Jing Zhang, Yanhui Gu, Xiaoli Liu, Ximin Rao, Guichuan Huang, Yao Ouyang

**Affiliations:** 1 Department of Pulmonary and Critical Care Medicine, The Affiliated Hospital of Zunyi Medical University, Zunyi, Guizhou 563000, China; 2 Department of Pulmonary and Critical Care Medicine, The Third Affiliated Hospital of Zunyi Medical University (The First People’s Hospital of Zunyi), Zunyi, Guizhou 563000, China

**Keywords:** meta analysis, non-small cell lung cancer, S100A4

## Abstract

**Background**: Numerous published studies have shown that S100A4 is frequently overexpressed in various human cancers. However, the association between S100A4 expression and prognosis or clinicopathological parameters in non-small cell lung cancer (NSCLC) remains unclear. Therefore, a meta-analysis was performed to identify the significance of S100A4 in NSCLC.

**Methods:** Systematic literature search was conducted using PubMed, Embase, Web of Science, the Cochrane Library, the Chinese National Knowledge Infrastructure database (CNKI), and the Wanfang database to obtain relevant articles. A combined hazard ratio (HR) and its corresponding 95% confidence interval (CI) were used to evaluate the association between S100A4 expression and prognosis in NSCLC patients. Pooled odds ratio (OR) and 95% CI were calculated to assess the association between S100A4 expression and clinicopathological features in NSCLC.

**Results**: NSCLC patients with overexpression of S100A4 had a worse prognosis than patients with low expression of S100A4 (HR = 1.77, 95% CI: 1.55–2.02, *P*<0.001). Additionally, overexpression of S100A4 was significantly correlated to patients’ age (OR = 0.67, 95% CI: 0.49–0.91, *P*=0.010), tumor differentiation (OR = 2.20, 95% CI: 1.69–2.85, *P*<0.001), lymph node metastasis (LNM) (OR = 3.70, 95% CI: 2.25–6.06, *P*<0.001), Tumor-Node-Metastasis (TNM) stage (OR = 3.08, 95% CI: 2.10–4.53, *P*<0.001), and pathological subtype (OR = 1.77, 95% CI: 1.09–2.88, *P*=0.020). However, there was no association between S100A4 expression and other clinicopathological features in NSCLC, including gender, tumor size, and smoking.

**Conclusion**: S100A4 overexpression was associated with tumor progression and poor prognosis in NSCLC patients. Hence, S100A4 might serve as a potential prognostic biomarker in NSCLC.

## Introduction

Lung cancer is the most commonly occurring cancer worldwide. Non-small cell lung cancer (NSCLC) accounts for more than 85% of all lung cancer cases, and NSCLC is classified into the following pathological subtypes: squamous cell carcinoma (SCC), adenocarcinoma (ADC), and large cell lung carcinoma [1]. Despite the great progress in the diagnosis and treatment of NSCLC, the overall survival (OS) rate of patients with NSCLC still remains poor. Hence, there is an urgent need to identify a novel biomarker for the detection, progression, and prognosis of NSCLC.

S100A4 (also known as mts1, 18A2, FSP1, p9Ka, pEL98), a member of the S100 family of calcium-binding proteins, is a small-molecular-weight (12 kDa) protein with two domains: an S100 domain and a calcium-binding domain [2,3]. S100A4 is present in the nucleus, cytoplasm, and extracellular space [4]. Overexpression of S100A4 has been reported in various malignancies, including pancreatic cancer [5], gastric cancer [6], colorectal cancer [7], esophageal cancer [8], and NSCLC [9]. Kimura et al. [10] reported that NSCLC patients with positive S100A4 expression had significantly shorter survival than the patients with negative S100A4 expression. Hu et al. [11] reported that S100A4 expression was related to lymph node metastasis (LNM) as well as Tumor-Node-Metastasis (TNM) stage. Jung et al. [12] reported that the histological type of NSCLC was significantly correlated with S100A4 expression. However, Li et al. [13] reported that no significant difference was noted on patients’ survival between S100A4 negative and S100A4 positive expression. Jung et al. [12] reported that S100A4 expression was not correlated with LNM, TNM stage or survival in patients with NSCLC. Hence, the prognostic value of S100A4 and the relationship between S100A4 expression and clinicopathological features still remain controversial. Although Bai et al. [14] concluded that overexpression of S100A4 expression was associated with poor prognosis in lung cancer, the number of studies included in their meta-analysis was relatively small, and the relationship between S100A4 expression and clinicopathological features in NSCLC was not investigated. Moreover, we identified a few errors in the extracted data used in Bai et al. [14] meta-analysis that should be verified and corrected. For instance, Matsubara et al. [15] reported that the patients with NSCLC with an overexpression of S100A4 had a worse OS than the NSCLC patients with low S100A4 expression (*P*=0.0269). In contrast, in Bai et al.’s [14] meta-analysis, the extracted data based on the survival curve (hazard ratio [HR] = 1.35, 95% confidence interval [CI]: 0.48–3.81) from Matsubara et al. [15] demonstrated that the expression of S100A4 was not associated with the prognosis of NSCLC. In addition, similar errors were also found in the data that Bai et al. [14] extracted from Qi et al. [16] and Chen et al. papers [17] based on the survival curve, which resulted in unreliable conclusion from Bai et al. meta-analysis [14]. Thus, we performed a new meta-analysis to identify the association of S100A4 expression with NSCLC prognosis and clinicopathological features.

## Materials and methods

### Literature search strategy

This meta-analysis was performed according to the Preferred Reporting Items for Systematic Reviews and Meta-Analyses (PRISMA) statement [18]. A systematic literature search was conducted through 5 March 2020, to identify relevant articles to be included in this meta-analysis and the search was carried out in the following databases: PubMed, Embase, Web of Science, the Cochrane Library, the Chinese National Knowledge Infrastructure database (CNKI), and the Wanfang database. The keywords used in databases search were as follows: ‘S100A4 or FSP1 or mts1 or 18A2 or pEL-98’ and ‘pulmonary or lung’ and ‘cancer or neoplasm or carcinoma or tumor or adenocarcinoma’. Literatures were initially screened by title and abstract for relevance, and then careful evaluation of the full-text articles was performed to identify relevant articles.

### Inclusion and exclusion criteria

The inclusion criteria were as follows: articles describing the relationship between S100A4 expression and survival outcome or clinicopathological parameters in patients with NSCLC; adequate data provided to calculate the odds ratio (OR), HR, and 95% CI; and the expression of S100A4 in NSCLC tissues determined by immunohistochemistry (IHC).

The exclusion criteria were as follows: articles pertaining to the case report, review, letter, and conference abstract; articles describing overlapping studies; or studies with insufficient data.

### Data extraction and literature quality assessment

Two reviewers (Jing Zhang and Guichuan Huang) independently extracted the relevant data from included studies. The following data were obtained from each included study: the name of the first author, publication year, country, language, sample size, detection method, clinicopathological parameters of NSCLC patients, HR, and corresponding 95% CI. If the required data were not provided directly but were provided as Kaplan–Meier curves, then the relevant data were obtained from graphical survival plots based on the described method [19]. Furthermore, the Newcastle–Ottawa Scale (NOS) was used to perform the quality assessment of each included study. Study with a NOS score ≥6 was considered as high quality.

### Statistical analysis

In this meta-analysis, all statistical analyses were performed using Stata-SE software (version 15.0). Also, HR and corresponding 95% CI were combined to evaluate the strength of the association between S100A4 expression and prognosis in NSCLC patients. Similarly, pooled OR and corresponding 95% CI were used to evaluate the association between S100A4 expression and clinicopathological parameters in NSCLC patients. Cochran’s Q (chi-square) test and *I^2^* statistics were applied to assess the heterogeneity among the studies. For the chi-square test, if *P*<0.05 or *I^2^* > 50%, it indicated the presence of heterogeneity, then a fixed-effect model was used. On the contrary, if *P*≥0.05 or *I^2^* ≤ 50%, a random-effect model was used. Begg’s test and Egger’s test were used to evaluate the potential publication bias. The trim and fill method was applied for correcting any publication bias identified in this meta-analysis [20]. A sensitivity analysis was performed to examine the robustness of results. A *P*-value of less than 0.05 was considered statistically significant.

## Results

### Study selection and characteristics

**Figure 1:**
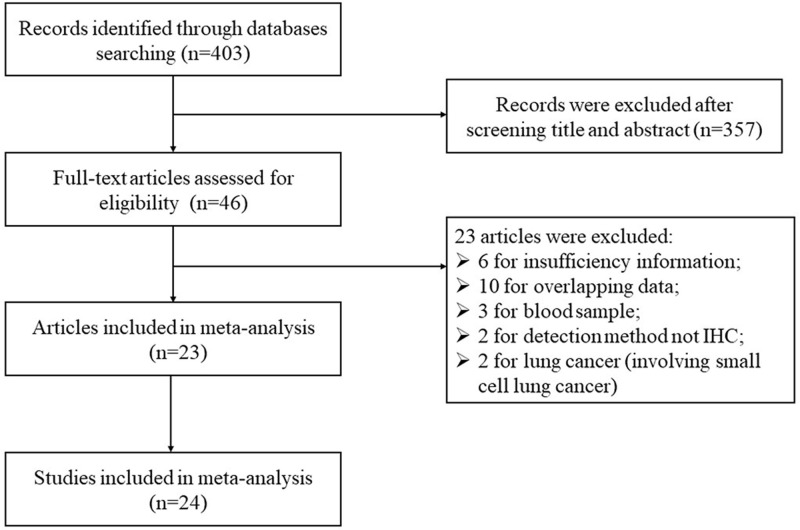
Flow diagram of literature search and selection process

The article selection process is presented in Figure 1. A total of 403 articles were retrieved from the databases search described above. After a careful initial evaluation of titles and abstracts, 46 potential articles were selected further for full-text screening. After reading the full-text of potential selected articles, a total of 23 articles involving 24 studies with 2307 patients were included in this meta-analysis [9–13,15–17,21–35].

The characteristics of the included studies published between 2000 and 2017 are presented in Table 1. Of the 24 studies, 18 studies were conducted in China, 2 in Switzerland, 2 in Japan, 1 in Korea, and 1 in America. Seventeen studies were published in Chinese, and seven studies were published in English. The expression of S100A4 in NSCLC patients was determined by IHC in all included studies. The NOS score for each included study in this meta-analysis was greater than or equal to 6, indicating high quality.

**Table 1:** Characteristics of included studies in the meta-analysis

Author	Year	Country	Language	Number of patients	Detection method	Gender: male (+/-), female (+/-)	Age: >50 (+/-), ≤50 (+/-)	Tumor size: >3 cm (+/-), ≤3 cm (+/-)	Differentiation: low (+/-), high, and moderate (+/-)	LNM: yes (+/-) no (+/-)	TNM stage: III+IV (+/-) I+II (+/-)	Distant metastasis: yes (+/-) no (+/-)	Pathological subtype: SCC (+/-) ADC (+/-)	Smoking history: yes (+/-) no (+/-)	Survival information	NOS scores
Kimura [10]	2000	Switzerland	English	135	IHC	NA	NA	NA	14/7 60/45	39/11 42/43	NA	3/0 78/54	NA	NA	OS (S)	8
Hu [11]	2005	China	Chinese	86	IHC	35/29 13/9	22/28 26/10	40/20 8/18	17/9 31/29	36/14 12/24	9/5 39/33	NA	32/27 16/11	NA	NA	6
Han [22]	2008	China	Chinese	130	IHC	62/21 32/15	57/25 37/11	NA	45/11 49/25	70/12 24/24	68/10 26/26	22/2 72/34	43/8 28/19	47/16 47/20	OS (M)	7
Matsubara [15]	2005	Switzerland	English	94	IHC	NA	NA	NA	NA	11/27 8/48	11/36 8/39	NA	NA	NA	OS (S)	8
Miyazaki [31]	2006	Japan	English	92	IHC	NA	NA	NA	NA	29/9 35/19	23/7 41/21	35/20 29/8	NA	NA	OS (S)	8
Qi [16]	2007	China	Chinese	116	IHC	49/33 15/19	38/44 26/8	53/23 11/29	NA	46/13 18/39	23/14 41/38	NA	29/26 32/24	NA	OS (S)	8
Chen [17]	2008	China	Chinese	41	IHC	17/8 12/4	16/7 13/5	21/2 8/10	15/4 14/8	18/2 11/10	16/0 13/12	NA	11/10 18/2	8/6 23/4	OS (S)	6
Liu [21]	2006	China	Chinese	47	IHC	10/3 18/16	NA	NA	12/5 16/14	21/8 7/11	NA	NA	NA	NA	NA	6
Sheng [9]	2006	China	Chinese	76	IHC	NA	NA	NA	22/14 21/19	30/8 9/29	38/14 8/16	NA	30/18 18/10	NA	NA	6
Tsuna [32]	2009	Japan	English	66	IHC	NA	NA	NA	NA	NA	NA	NA	NA	NA	OS (S)	7
Tsuna [32]	2009	Japan	English	104	IHC	NA	NA	NA	NA	NA	NA	NA	NA	NA	OS (S)	7
Lin [33]	2009	China	Chinese	91	IHC	46/21 21/3	38/12 29/12	50/21 17/3	NA	32/6 35/18	23/3 44/21	NA	33/18 34/6	NA	NA	7
Qin [23]	2009	China	Chinese	130	IHC	56/27 34/13	49/33 41/7	NA	48/8 42/32	68/14 22/26	65/13 25/27	21/3 69/37	NA	46/17 44/23	OS (M)	7
Xu [24]	2009	China	Chinese	120	IHC	48/28 29/15	44/35 33/8	NA	33/11 36/32	18/31 59/12	55/25 22/18	NA	24/25 34/13	NA	NA	6
Jung [12]	2010	Korea	English	67	IHC	43/10 13/1	18/6 38/5	NA	NA	25/4 30/7	16/3 40/8	2/0 54/11	27/10 29/1	NA	OS	8
Yang [25]	2010	China	Chinese	90	IHC	38/13 31/8	22/9 47/12	NA	39/4 30/17	51/10 18/11	45/4 24/17	NA	33/11 36/10	NA	NA	7
Li [13]	2011	China	Chinese	79	IHC	13/15 26/25	22/21 17/19	NA	NA	27/18 12/22	28/16 14/21	NA	NA	17/10 22/30	OS (S)	7
Chen [26]	2012	China	Chinese	112	IHC	38/38 22/14	22/16 38/36	32/35 28/17	18/15 42/37	NA	NA	10/10 50/42	24/13 38/30	NA	OS (S)	6
Zhang [34]	2013	China	English	204	IHC	114/59 23/8	67/29 70/38	NA	38/17 99/50	59/19 78/48	43/16 94/51	NA	NA	110/55 27/12	OS (M)	8
Zhang [27]	2013	China	Chinese	89	IHC	45/7 30/7	39/8 36/6	NA	8/0 67/14	55/2 20/12	55/3 20/11	56/2 19/12	26/6 40/10	NA	OS (M)	7
Yang [28]	2014	China	Chinese	67	IHC	30/11 19/7	29/13 20/5	NA	16/1 33/17	34/5 15/13	NA	NA	21/12 25/2	NA	NA	7
Liu [29]	2015	China	Chinese	84	IHC	31/11 27/15	35/15 23/11	26/12 32/14	26/1 32/25	35/1 23/25	37/1 21/25	NA	NA	NA	OS (S)	7
Stewart [35]	2016	America	English	81	IHC	NA	NA	NA	NA	NA	NA	NA	6/94 17/64	NA	OS	8
Chen [30]	2017	China	Chinese	106	IHC	35/19 34/18	24/15 45/22	NA	NA	31/5 38/32	33/10 36/27	NA	11/15 58/22	25/16 44/21	OS (M)	7

Abbreviations: M, multivariate analysis; NA, not available; S, survival curve.

### Relationship between S100A4 expression and OS in NSCLC

**Figure 2:**
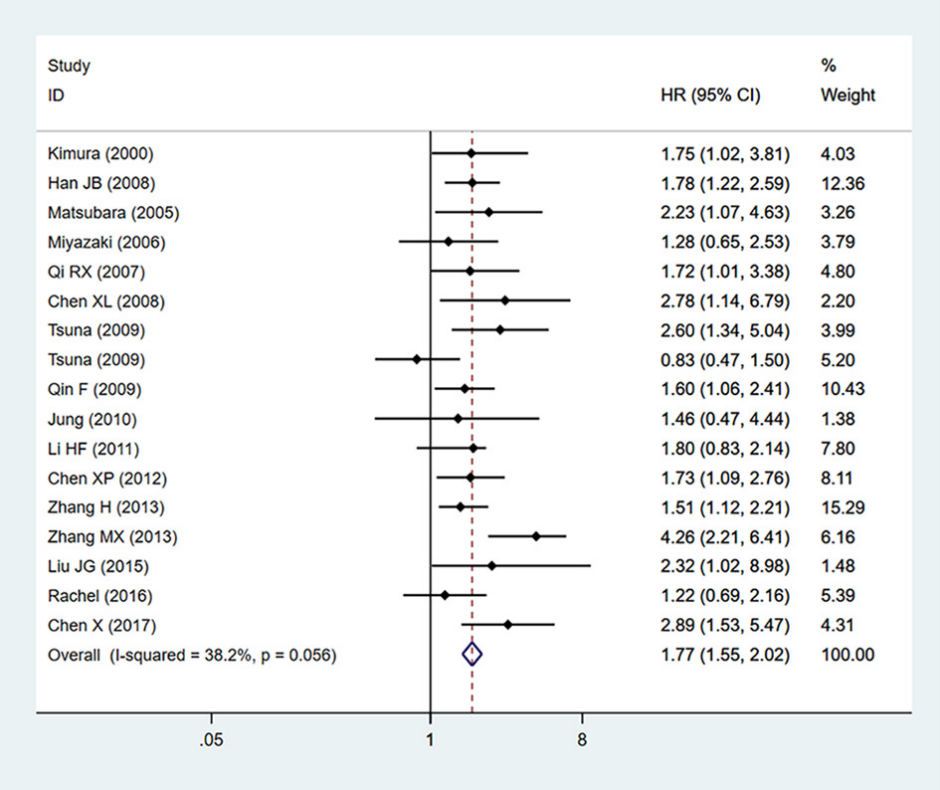
Forest plot for the association between S100A4 expression and OS

Of the 24 included studies in this meta-analysis, 17 studies including 1730 patients reported data on S100A4 expression and OS in NSCLC. As shown in Figure 2, no significant heterogeneity (*P*=0.056, *I^2^*= 38.2%) was found, and thus, a fixed-effect model was utilized for the combined HR and 95% CI. The results indicated NSCLC patients with overexpression of S100A4 had a worse OS than patients with low expression of S100A4 (HR = 1.77, 95% CI: 1.55–2.02, *P*<0.001).

### Relationship between S100A4 expression and clinicopathological parameters in NSCLC

**Figure 3:**
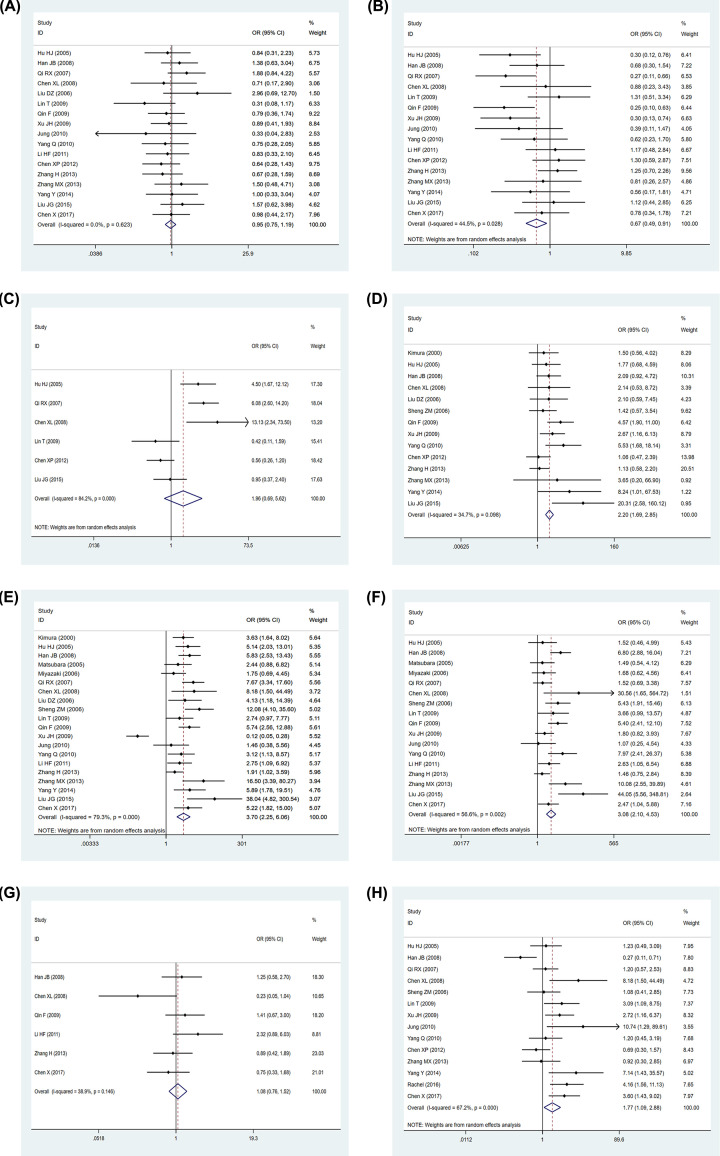
Forest plot for the association between S100A4 expression and clinicopathological features Forest plot for the association between S100A4 expression and clinicopathological features, including (**A**) gender, (**B**) age, (**C**) tumor size, (**D**) tumor differentiation, (**E**) LNM, (**F**) TNM stage, (**G**) smoking, (**H**) pathological subtype.

The relationship between S100A4 expression and clinicopathological parameters in NSCLC patients is illustrated in Figure 3 and Table 2. Overexpression of S100A4 was correlated with age (> 50 vs. ≤50) (*P*=0.010, OR = 0.67, 95% CI: 0.49–0.91, *I^2^* = 44.5%), tumor differentiation (poorly vs. well and moderately) (*P*<0.001, OR = 2.20, 95% CI: 1.69–2.85, *I^2^* = 34.7%), LNM (yes vs. no) (*P*<0.001, OR = 3.70, 95% CI: 2.25–6.06, *I^2^* = 79.3%), TNM stage (III/IV vs. I/II) (*P*<0.001, OR = 3.08, 95% CI: 2.10–4.53, *I^2^* = 56.6%), and pathological subtype (ADC vs. SCC) (*P*=0.020, OR = 1.77, 95% CI: 1.09–2.88, *I^2^* = 67.2%). However, no significant association was identified between overexpression expression of S100A4 and other clinicopathological parameters, including gender (male vs. female) (*P*=0.641, OR = 0.95, 95% CI: 0.75–1.19, *I^2^*= 0.0%), tumor size (>3 vs. ≤3 cm) (*P*=0.209, OR = 1.96, 95% CI: 0.69–5.62, *I^2^*= 84.2%), and smoking (yes vs. no) (*P*=0.673, OR = 1.08, 95% CI: 0.76–1.52, *I^2^*= 38.9%).

**Table 2:** The correlation between S100A4 expression and clinicopathological features

Clinicopathological features	Number of studies	Number of patients	*P*-value	OR (95% CI)	Heterogeneity		Model
					*I^2^* (%)	*P*_*Het*_	
Gender	17	1659	0.641	0.95 (0.75–1.19)	0.0	0.623	fixed
Age	16	1612	0.010	0.67 (0.49–0.91)	44.5	0.028	random
Tumor size	6	530	0.209	1.96 (0.69–5.62)	84.2	<0.001	random
Differentiation	14	1411	<0.001	2.20 (1.69–2.85)	34.7	0.098	fixed
LNM	20	1944	<0.001	3.70 (2.25–6.06)	79.3	<0.001	random
TNM stage	17	1695	<0.001	3.08 (2.10–4.53)	56.6	0.002	random
Smoking	6	690	0.673	1.08 (0.76–1.52)	38.9	0.146	fixed
Pathological subtype	14	1272	0.020	1.77 (1.09–2.88)	67.2	<0.001	random

Owing to significant heterogeneity that existed in age analysis (*I^2^* = 44.5%, *P_Het_*=0.03), a random-effect model was used. We found that the heterogeneity was obviously reduced (*I^2^* = 37.8%, *P_Het_*=0.07) without a change in combined outcome (OR = 0.61, 95% CI: 0.48–0.78) after removing Zhang et al. [34] study data from the pooled analysis. The subgroup analysis of LNM was performed based on publication year, sample size, and ethnicity because of significant heterogeneity. As shown in Supplementary Table S1, in the subgroup analysis of sample size, overexpression of S100A4 was related to LNM both in large (≥90) and small sample size groups (<90). Likewise, similar outcomes were observed in the subgroup analysis based on publication year and ethnicity. As all subgroup analyses of LNM based on sample size, ethnicity, and publication year indicated heterogeneity, the S100A4 expression in relation to the heterogeneity of LNM was not likely caused by publication year, sample size, and ethnicity.

The subgroup analyses of the TNM stage (Supplementary Table S2) and pathological subtype (Supplementary Table S3) were also conducted to identify the sources of heterogeneity on the basis of publication year, sample size, and ethnicity. However, heterogeneity remained practically unchanged, indicating that heterogeneity probably did not result from publication year, sample size, and ethnicity.

### Sensitivity analysis

To test whether a single study affected the pooled HR or OR, a sensitivity analysis was performed. After removing individual study, the corresponding pooled HR and OR was not significantly changed, indicating the robustness of our meta-analysis results (data not shown).

### Publication bias

Begg’s test and Egger’s test were conducted to evaluate the publication bias in this meta-analysis. As shown in Table 3, no publication bias was observed for the association between S100A4 expression and OS, gender, age, tumor size, LNM, TNM stage, smoking, or pathological subtype. However, publication bias was found in the association between S100A4 expression and tumor differentiation in patients with NSCLC (*P*=0.044 for Begg’s test; *P*=0.013 for Egger’s test) (Table 3). After applying the ‘trim and fill’ method to adjust effects for publication bias, the association between S100A4 expression and tumor differentiation was still found significant (fixed-effect model: OR = 1.89, 95% CI: 1.45–5.47, *P*<0.001; random-effect model: OR = 1.98, 95% CI:1.34–2.91, *P*=0.001), suggesting that this observed publication bias may not influence the pooled results.

**Table 3:** Publication bias by Begg’s test and Egger’s test

Clinicopathological features	Number of studies	Estimates	Begg’s test (*P*-value)	Egger’s test (*P*-value)	Publication bias
OS	17	HR + 95% CI	0.343	0.440	not significant
Gender	17	OR + 95% CI	0.837	0.514	not significant
Age	16	OR + 95% CI	0.260	0.181	not significant
Tumor size	6	OR + 95% CI	0.707	0.615	not significant
Differentiation	14	OR + 95% CI	0.044	0.013	significant
LNM	20	OR + 95% CI	0.206	0.130	not significant
TNM stage	17	OR + 95% CI	0.096	0.077	not significant
Smoking	6	OR + 95% CI	0.452	0.297	not significant
Pathological subtype	14	OR + 95% CI	0.155	0.064	not significant

## Discussion

S100A4 belongs to the S100 family of calcium-binding proteins, and this multifunctional protein is presented in the cytoplasm, nucleus, and extracellular space [36]. Previous studies have shown that S100A4 is involved in a series of intracellular and extracellular biological functions and activities, including regulation of angiogenesis, cell motility, cell differentiation, and invasion [37].

Numerous studies have indicated that S100A4 is associated with tumor progression. Liu et al. [38] reported that S100A4 overexpression was significantly correlated with the poor OS, tumor location, LNM, TNM stage, and tumor depth in colorectal cancer. A study by Ling et al. [39] indicated that S100A4 overexpression was significantly associated with tumor grade, stage, metastasis, invasion, and relapse as well as worse OS in patients with gastric cancer. Although a meta-analysis performed by Bai et al. [14] has shown that overexpression of S100A4 was associated with poor prognosis in lung cancer, the number of included studies was relatively small and the relationship between S100A4 expression and clinicopathological features in NSCLC was not investigated. Therefore, we conducted this meta-analysis.

A total of 23 articles involving 24 studies with 2307 patients were included. Of the 17 studies reporting datasets of S100A4 expression and OS in NSCLC, 5 studies results indicated that S100A4 expression was not associated with prognosis in NSCLC. However, pooled results of this meta-analysis indicated that S100A4 overexpression was significantly associated with poor OS in NSCLC patients. In addition, present meta-analysis results indicated that overexpression expression of S100A4 was associated with clinicopathological characteristics, including patient’s age, tumor differentiation, LNM, TNM stage, and pathological subtype. However, no significant relationship was found between S100A4 expression and gender, tumor size, and smoking.

Of the 16 studies reporting datasets of S100A4 expression and patients’ age in NSCLC, four studies results suggested a negative association between S100A4 expression and NSCLC patients’ age. The pooled results of this meta-analysis also proved this negative association between NSCLC patient’s age and S100A4 expression. Young-age patients with NSCLC had a greater extent of malignancy and mortality rate than elderly patients [40], and most of the young-aged lung cancer patients had ADC [41], all of which may contribute to the negative relationship between the S100A4 expression and age. Kimura et al. [10] acknowledged that S100A4 expression was correlated with LNM in NSCLC. However, another study results indicated that no relationship was observed between S100A4 expression and LNM [12]. In the present meta-analysis, the results indicated that the expression of S100A4 had a significant correlation with LNM in NSCLC. Likewise, 8 of 17 included studies showed that S100A4 was not associated with TNM stage. However, pooled results of present meta-analysis demonstrated that S100A4 was positively correlated with TNM stage. S100A4 is a promoting protein for epithelial–mesenchymal transition and plays an important role in facilitating tumor invasion and metastasis [42]. Hence, it is not hard to understand the association between S100A4 and LNM as well as the TNM stage. Five of 14 included studies in the meta-analysis indicated that tumor differentiation was correlated with S100A4 expression. The combined outcomes in the present meta-analysis also demonstrated that a significant association was observed between S100A4 expression and tumor differentiation in NSCLC. Additionally, it was also found that S100A4 expression was also associated with pathological subtype in NSCLC and S100A4 expression showed a higher positivity in ADC than in SCC. Owing to significant heterogeneity existed in the analysis of LNM, TNM stage, and pathological subtype, subgroup analysis was conducted based on publication year, sample size, and ethnicity. However, the sources of heterogeneity were not established. Therefore, more high-quality and large-sample size studies are required.

There are several limitations that should be considered in the present meta-analysis. First, due to a lack of original data for some studies, HRs were extracted from the Kaplan–Meier survival curves. Second, the cut-off values for defining S100A4 expression are different among the included studies, which may result in heterogeneity. Third, obvious heterogeneity was observed in the analysis of age, tumor size, LNM, TNM stage, and pathological subtype, which may reduce the reliability of results. Fourth, publication bias was found in the analysis of tumor differentiation, although results remained unchanged after adjustment by the trim and fill method. Finally, most of the included studies in this meta-analysis were retrospective studies, which caused the selection bias. Hence, the results should be interpreted cautiously.

## Conclusion

Overexpression of S100A4 is associated with clinicopathological features of NSCLC, including age, differentiation, LNM, TNM stage, pathological subtype, with a tendency for poor prognosis. Hence, S100A4 may serve as a potential prognostic biomarker for patients with NSCLC.

## Supplementary Material

Supplementary Tables S1-S3

## Abbreviations

ADC: adenocarcinoma
CI: confidence interval
HR: hazard ratio
IHC: immunohistochemistry
LNM: lymph node metastasis
NOS: Newcastle–Ottawa Scale
NSCLC: non-small cell lung cancer
OR: odds ratio
OS: overall survival
SCC: squamous cell carcinoma
TNM: tumor-node-metastasis
