# Supplementary material for: Clinicopathological and prognostic value of S100A4 expression in non-small cell lung cancer: a meta-analysis
Source: Biosci Rep. 2020 Jul 30;40(7):BSR20201710. doi: 10.1042/BSR20201710 (PMC7396424; doi:10.1042/BSR20201710)
Supplement: Supplementary Tables S1-S3 [file BSR-2020-1710_supp.pdf]

Supplementary Table 1 Results of subgroup analysis of LNM by publication year, sample size, and ethnicity

Subgroup analysis	No.of studies	No.of patients	***P* value**	**OR (95%CI)**	**Heterogeneity**	
					I^2(%)	P_{Het}
Total	20	1944	<0.001	3.70 (2.25-6.06)	79.3	<0.001
Publication year						
<2009	9	817	<0.001	4.66 (3.15-6.88)	29.1	0.186
≥2009	11	1127	0.007	3.11 (1.36-7.08)	85.9	<0.001
Sample size						
<90	9	636	<0.001	2.61 (1.31-5.21)	85.5	<0.001
≥90	11	1308	0.006	5.82 (3.35-10.10)	42.1	0.086
Ethnicity						
Chinese	16	1556	<0.001	4.28 (2.31-7.91)	83.0	<0.001
Non-Chinese	4	388	<0.001	2.42 (1.49-3.93)	0.0	0.568

Abbreviations: LNM, lymph node metastasis; OR, odds ratio.

Supplementary Table 2 Results of subgroup analysis of TNM stage by publication year, sample size, and ethnicity

Subgroup analysis	No.of studies	No.of patients	*P* value	OR (95%CI)	Heterogeneity	
					I^2(%)	P_{Het}
Total	17	1695	<0.001	3.08 (2.10-4.53)	56.6	0.002
Publication year						
<2009	7	635	0.001	2.73 (1.47-5.08)	56.9	0.030
≥2009	10	1060	<0.001	3.39 (2.01-5.70)	60.5	0.007
Sample size						
<90	7	522	0.001	4.58 (1.93-10.83)	63.0	0.013
≥90	10	1173	<0.001	2.64 (1.75-3.99)	52.5	0.026
Ethnicity						
Chinese	14	1442	<0.001	3.63 (2.36-5.60)	59.4	0.002
Non-Chinese	3	253	0.239	1.47 (0.77-2.78)	0.0	0.878

Abbreviation: OR, odds ratio.

Supplementary Table 3 Results of subgroup analysis of Pathological subtype by publication year, sample size, and ethnicity

**Subgroup analysis**	**No.of studies**	**No.of patients**	***P* value**	**OR (95%CI)**	**Heterogeneity**	
					**I^2(%)**	**P_{Het}**
Total	14	1272	0.020	1.77 (1.09-2.88)	67.2	<0.001
Publication year						
<2009	5	449	0.805	1.11 (0.49-2.51)	70.2	0.009
≥2009	9	823	0.003	2.30 (1.33-3.99)	57.8	0.015
Sample size						
<90	7	507	0.011	2.54 (1.23-5.22)	57.7	0.028
≥90	7	765	0.383	1.34 (0.69-2.60)	73.8	0.001
Ethnicity						
Chinese	12	1124	0.093	1.52 (0.93-2.49)	65.6	0.001
Non-Chinese	2	148	<0.001	4.92 (2.02-12.01)	0.0	0.421

Abbreviation: OR, odds ratio.
